# Antidepressant-like activity of tramadol in mice

**DOI:** 10.4103/0019-5545.39760

**Published:** 2008

**Authors:** Bhupinder Singh Kalra, Vandana Tayal, Shalini Chawla

**Affiliations:** Department of Pharmacology, Maulana Azad Medical College, New Delhi, India

**Keywords:** Antidepressant, forced swim test, tramadol

## Abstract

**Objective:**

To evaluate antidepressant-like effect of tramadol in mice.

**Materials and Methods:**

Tramadol was administered at three different doses (10, 20, and 40 mg/kg, i.p.) once daily for 7 days to Swiss albino mice of either sex. The immobility period of control and drug-treated mice was recorded in forced swim test (FST). The antidepressant effect of tramadol was compared to that of fluoxetine (20 mg/kg, i.p.), administered for seven successive days.

**Results:**

Tramadol produced significant antidepressant effect at all the three (10, 20, and 40 mg/kg) doses, as indicated by reduction in immobility times of drug-treated mice compared to control mice. The efficacy of tramadol at doses of 10 and 20 mg/kg was comparable to that of fluoxetine, but antidepressant activity in animals administered with tramadol 40 mg/kg was significantly less as compared to fluoxetine-pretreated mice.

**Conclusion:**

The results of the present study indicate antidepressant-like activity of tramadol.

## INTRODUCTION

Tramadol is a synthetic centrally acting opioid analgesic used mainly for the treatment of moderate-to-severe pain.[1] It is a weak *μ* opioid receptor agonist, and it also produces analgesia by inhibiting uptake of norepinephrine and serotonin.[1] Tramadol causes activation of both systems mainly involved in inhibition of pain, i.e., the opioid and the descending monoaminergic pain-modulating pathways.

There is a large body of evidence to suggest that the analgesic action of tramadol is mainly related to central monoaminergic mechanism rather than opioid receptor pathway. It has also been observed that tramadol-induced analgesia is blocked by α_2_ adrenergic receptor antagonist Yohimbine.[2]

*In vitro* studies have shown that tramadol effectively inhibits reuptake of monoamines.[3] It has also been established that tramadol inhibits reuptake of serotonin in the raphe nucleus.[4] Antidepressants mainly act by inhibiting norepinephrine/serotonin reuptake; tramadol by virtue of its property of blocking monoaminergic reuptake may act as an antidepressant. Also, tramadol bears a close structural similarity to antidepressant Venlafaxine and thus shares a number of its molecular and pharmacological features.[5]

In a study conducted in mice using an experimental model, it was seen that tramadol exhibits antidepressant activity.[1] Another study in rats showed that tramadol led to decrease in the number of failures to avoid or escape aversive stimuli (shock) in *learned helplessness* model.[6] Few documented clinical reports have also indicated the possibility of antidepressant effect of tramadol. In one case report, a case of severe suicidal ideation rapidly resolved with intramuscular tramadol.[7] Tramadol monotherapy was also reported to be effective in a case of refractory major depression.[8]

Hence this study was undertaken with the objective of studying the antidepressant-like activity of tramadol in animal model of depression.

## MATERIALS AND METHODS

This study was conducted in the Department of Pharmacology, Maulana Azad Medical College, after getting approval from the Institutional Review Board and Animal Ethical Committee. Animals were procured from central animal house and were kept in an air-conditioned environment. After procurement till the beginning of study, an interval of 1 week was scheduled for acclimatization of animals. Mice of either sex weighing 20-25 g were taken for the study. They were provided with normal food pellet diet with water *ad libitum.*

### Study design

Animals were divided into five groups, and each group comprised of eight mice. Group 1 (control group) was pretreated with normal saline (0.1 ml/10 g). Groups 2, 3 and 4 were pretreated with three different doses (10, 20, and 40 mg/kg) of tramadol for 7 days [Table 1]. Group 5 was pretreated with fluoxetine at the dose of 20 mg/kg intraperitonially (0.1 ml/10 g) for 7 days. Tramadol and fluoxetine were dissolved in normal saline.

**Table 1:** Effect of different doses of tramadol on immobility period in forced swim test (*n* = 8)

Groups	Treatment with dose(mg/kg)	Immobility period (s) Mean ± S
1	Control (normal saline), 1 ml/100g	206.2 ± 8.0
2	Tramadol, 10 mg/kg	88.1 ± 5.5*
3	Tramadol, 20 mg/kg	95 ± 22.5*
4	Tramadol, 40 mg/kg	146.1 ± 8.5*,α
5	Fluoxetine, 20 mg/kg	83.6 ± 6.5*

* *P* < 0.001 as compared to control,

α *P* < 0.001 as compared to fluoxetine

## ANIMALS

Swiss albino mice of either sex (3 months old) were used in the present study.

### Animal model for testing antidepressant activity

#### Forced swim test:

This animal model is based on the principle that forcing mice to swim in restricted space from which they cannot escape leads to a characteristic behavior of immobility. This behavior reflects a state of despair, which can be reduced by several agents that are therapeutically effective in human depression.

Test drugs were administered intraperitonially for 7 days. On day 7, drugs were administered to mice 40 min prior to testing. Mice were individually forced to swim inside vertical plexiglass cylinder (height, 25 cm; diameter, 10 cm) containing water column of 15 cm height.[9] After an initial 2-min period of vigorous activity, usually each animal assumes a typical immobile posture. A mouse was considered immobile when it remained floating in the water without struggling, making only minimum movements of its limbs necessary to keep its head above water. The total duration of immobility was recorded during the next 4 min of the total 6 min of the duration of the test.[10] Durations of immobility period were compared with those of control and fluoxetine group. All results are expressed as mean ± SD. All the groups were analyzed using one-way ANOVA. *P* < 0.05 was considered significant.

## RESULTS

### Effect on immobility period in different groups

Mean duration of immobility in the control group was observed to be 206.25 ± 8.0 s, whereas it was 83.6 ± 6.5 s in the group pretreated with fluoxetine. The decrease in immobility period in the group pretreated with fluoxetine as compared to control was highly significant (*P* < 0.001). In the groups pretreated with three different doses of tramadol for 7 days, the immobility period was 88.1 ± 5.5 s for group 2, 95 ± 22.5 s for group 3, and 146.1 ± 8.5 s for group 4 [Table 1]. Tramadol at all three doses significantly decreased immobility period as compared to control mice (*P* < 0.001). Decrease in immobility period in the groups pretreated with tramadol (10 and 20 mg/kg) was not found be significant as compared to fluoxetine-pretreated mice (group 5), but antidepressant activity in animals administered with tramadol 40 mg/kg (group 4) was significantly less as compared to fluoxetine-pretreated mice.

## DISCUSSION

In this study, antidepressant effect of tramadol was evaluated in the forced swim test, a standard animal model predictive of antidepressant activity. Tramadol produced significant antidepressant effect at all the three doses. The antidepressant effect of tramadol at doses of 10 and 20 mg/kg was comparable with that of fluoxetine.

Similar findings were observed in an earlier study.[1] In our study, we found significant decrease in antidepressant activity of tramadol at higher dose (40 mg/kg) as compared to fluoxetine, but this decrease in antidepressant activity was not significant as compared to that in control mice. This finding in our study was in contrast to an earlier study in which antidepressant activity consistently increased with increase in dose.[1]

Antidepressants (selective serotonin reuptake inhibitors; Venlafaxine), by virtue of their property of mood elevation and increasing the level of serotonin and consequently causing inhibition of release of transmitters carrying the pain sensation from nerve endings, are efficacious in chronic pain as an adjunctive treatment.[1,5,11] Similarly, it could be inferred from our study that tramadol by acting through similar mechanism (inhibition of reuptake of monoamines leading to spinal inhibition of pain) might add a mood-elevation component to its analgesic effect. More preclinical studies in different antidepressant models are needed to corroborate our observations.
